# A cognitive approach to better understand foraging strategies of the adult domestic hen

**DOI:** 10.1038/s41598-024-70093-3

**Published:** 2024-08-20

**Authors:** R. Degrande, F. Cornilleau, P. Jardat, V. H. B. Ferreira, L. Lansade, L. Calandreau

**Affiliations:** CNRS, IFCE, INRAE, PRC (Physiologie de la Reproduction et des Comportements), Université de Tours, 37380 Nouzilly, Indre-et-Loire France

**Keywords:** Animal behaviour, Cognitive neuroscience

## Abstract

Foraging is known to be one of the most important activities in the behavioral budget of chickens. However, how these animals adapt different foraging strategies to diverse environmental variations is currently poorly understood. To gain further insight into this matter, in the present study, hens were submitted to the sloped-tubes task. In this task, the experimenter can manipulate the information that enables the hens to find a food reward (visible or not), placed in one of two hollow tubes. First, 12 hens were tested under free-choice conditions (no penalty for exhaustive searching in both tubes). Under these conditions, the hens adopted a non-random, side-biased strategy when the food location was not directly visible. Then, we divided the hens in two cohorts of equal size to study deeper the hens’ foraging strategy when faced (1) with a different container, or (2) with a restrictive environmental constraint under forced-choice conditions (no food reward if the unbaited tube is visited first). This latter constraint increased the risk of the hen not receiving food. A change in the containers didn’t modify the search behavior of the hens. However, in forced-choice conditions when the location of the food was not directly visible, four out of six hens learned to choose by exclusion. We conclude that hens can selectively adapt their foraging strategy to the point of adopting an exclusion performance, depending on available information and environmental constraints (high or low risk).

## Introduction

The domestic hen is the most common farmed animal around the world, and also the second most studied bird species in animal cognitive sciences^1^. Recently, interest in combining fundamental and applied knowledge of farm animal behavior and cognition has intensified, with the aim of improving their quality of life and welfare^2–4^. One of the most important behavioral activities of domestic fowl is foraging, i.e., searching for food by exploring substrates, with birds devoting up to 60% of their behavioral time budget to this activity^5,6^. For example, it has been demonstrated that chickens are motivated to work for food (contrafreeloading) and that the frequency of this behavior depends on individual parameters^7,8^. Foraging activity might also be an important way of improving welfare in farmed chickens, as the facilitation of foraging behaviors have been found to reduce the development of damaging behaviors such as feather pecking behaviors^9^, by fulfilling and redirecting exploratory motivation towards the environment^10,11^. But still, the strategies underlying chicken’s foraging behaviors still deserve consideration.

As exploratory foragers, chickens might search randomly for food; however, depending on the environmental constraints, they might be able to adapt and apply different, optimal strategies. Studies comparing the Red junglefowl, the living ancestor of domestic fowl, and the domestic hen (White Leghorn breed) have revealed that their foraging strategies are far from random. Birds employ different strategies to optimize their foraging: Red junglefowls prefer to contrafreeload (i.e., make foraging efforts to obtain their food, even though the same food is freely available), and presumably obtain more environmental information by exploring. Instead, domestic hens tend to contrafreeload less and maximize their direct gains. In both cases, this preference seems to be driven by long-term environmental constraints about food availability and uncertainty levels^12–16^.

Domestic chickens have also been found to adapt their foraging to different environmental cues^17,18^. For example, in a spatial task, where birds had to retrieve the location of a food reward hidden in a cup among seven other unbaited cups, they were able to retrieve the reward by using different spatial information. The hens were able to use cues close to the goal, called proximal cues, such as the color of the cup. But they were also able to use the relative positions of cues far from the goal, called distal cues, and to use their relational spatial memory when the proximal cues were absent. Combined, these results suggest that chickens are able to use selective strategies when searching for food, depending on environmental conditions.

Studies have revealed that other bird species, such as raven, keas^19^ or western scrub-jays^20^, can adapt their foraging strategies to the level of information available. In these studies, the main experiment involves hiding a food reward in one of two (or more) containers, while controlling the information available about the location of the food, for example through visual information. With this methodology, the modalities of foraging behaviors can be tested with a more cognitive approach, making it possible to study, for example, metacognitive control (i.e., the increased search for information when the uncertainty level is higher) or reasoning strategies. An example of the latter is inference by exclusion, which is the ability to select a correct alternative among other incorrect alternatives, by avoiding the incorrect alternatives^21^. For example, if the individual is faced with two containers and sees that the food reward is not in one of them, they will choose the other one, even though they did not see the location of the reward in a specific container. While this search strategy has already been observed in several bird species (mainly in corvids and parrots^22–26^; in ground-hornbills^27^, skuas^28^, pigeons^29^), the foraging behavior of chickens under different levels of information about the food location, and in different environmental constraints, has not yet been studied.

This study aimed at investigating how domestic hens adapt their foraging strategy depending on the information available to reach the location of a food reward, and the influence of environmental changes and constraints. We adapted the sloped-tubes task^19,21^ in which different configurations of two straight hollow tubes were presented, with either the inside of one or both tubes (visible content) or the side of one or both tubes (non-visible content) facing the animal. We were interested in the foraging strategy (which tube was visited first) of the hens depending on the configuration of the tubes. These different configurations lead to either complete, partial, or no visual information about the location of the reward for the hen.

First, the hens were tested under free-choice conditions where they were free to investigate both tubes with no penalty for exhaustive searching. Under these conditions, the food reward was always available even if their first choice was the unbaited tube. We hypothesized that the hens would move directly towards the food reward when they could see it, and choose randomly when they had no visual information about the location of the reward. Indeed, domestic birds are likely to develop passive and non-goal oriented behaviors when they have no information about the goal (see pheasant chicks during problem solving tasks)^30^. However, the use of an alternative strategy could not be ruled out, as other studies have demonstrated that hens can favor contrafreeloading even if an identical food reward is freely available^31^. For example, a well-known choice strategy in the sloped-tubes task is exclusion, when the bird uses the information of no reward in one tube (content visible) to select the other tube (content not visible), even if they cannot directly see the reward in it^19,21^. Due to contradictory hypotheses in the literature, we made no assumptions or predictions about a potential search strategy in configurations where hens cannot see the food reward directly.

Then, to study in greater depth the adaptation of hens’ foraging strategies to environmental changes and constraints, we divided the group in two cohorts of equal size. With the first cohort, we tested their behavior when faced with a change in food containers: we tested the hens’ foraging behavior under similar free-choice conditions but using another container, i.e., a square box, with different features (size, depth and openings). With the second cohort, we tested their search behavior under a change in environmental constraints: we tested the hens’ foraging behavior under forced-choice conditions. In these conditions, the reward was no longer available if the hen did not first investigate the baited tube, and the risk of not obtaining the reward was significantly higher than under free-choice conditions.

## Methods

### Ethical approval

This experimental procedure was approved by the Val de Loire Ethics Committee (approval no CE19—2020-0601-1, CEEA VdL, France). Animal care and experimental treatments complied with the French and European guidelines for housing and care of animals used for scientific purposes (European Union Directive 2010/63/AU). This study was reported in accordance with ARRIVE guidelines.

### Subjects

Twelve adult laying hens (Isa Brown), aged 1.5 to 3 years, were included in the procedure. The hens were maintained at the Pôle d’Expérimentation Avicole de Tours, where the experiment took place (UE PEAT, INRAE, 2018. Experimental Poultry Facility, 10.15454/1.5572326250887292E12). Hens were housed in a wood-chip littered barn (25 m^2^) with daylight source indoor and equipped with nesting boxes and perches, and had all-day access to an outside enclosure (about 30 m^2^) enriched with perches. Water was provided ad libitum, and food was delivered at will in large trough once the experiments of each testing day were completed, until the start of the dark phase, i.e., from mid-day to 8 pm. Birds were kept in a stable social group of 20 individuals on a 6 am to 8 pm daylight cycle. All experiments took place between 9 am and 1 pm.

As youngs, the subjects had been caught from a large group of chicks, at sexual maturity, and were habituated to their new environment and to experimenter’s manipulation during around 2 months before the beginning of any experiment. Previous to this experiment, the subjects had been involved in a pre-experiment in a totally different apparatus (same pre-experiment for all the individuals) with no similarity to the experiment described in this manuscript, until two months before the beginning of the experiment.

### Experimental set-up and general apparatus operative

**Figure 1:**
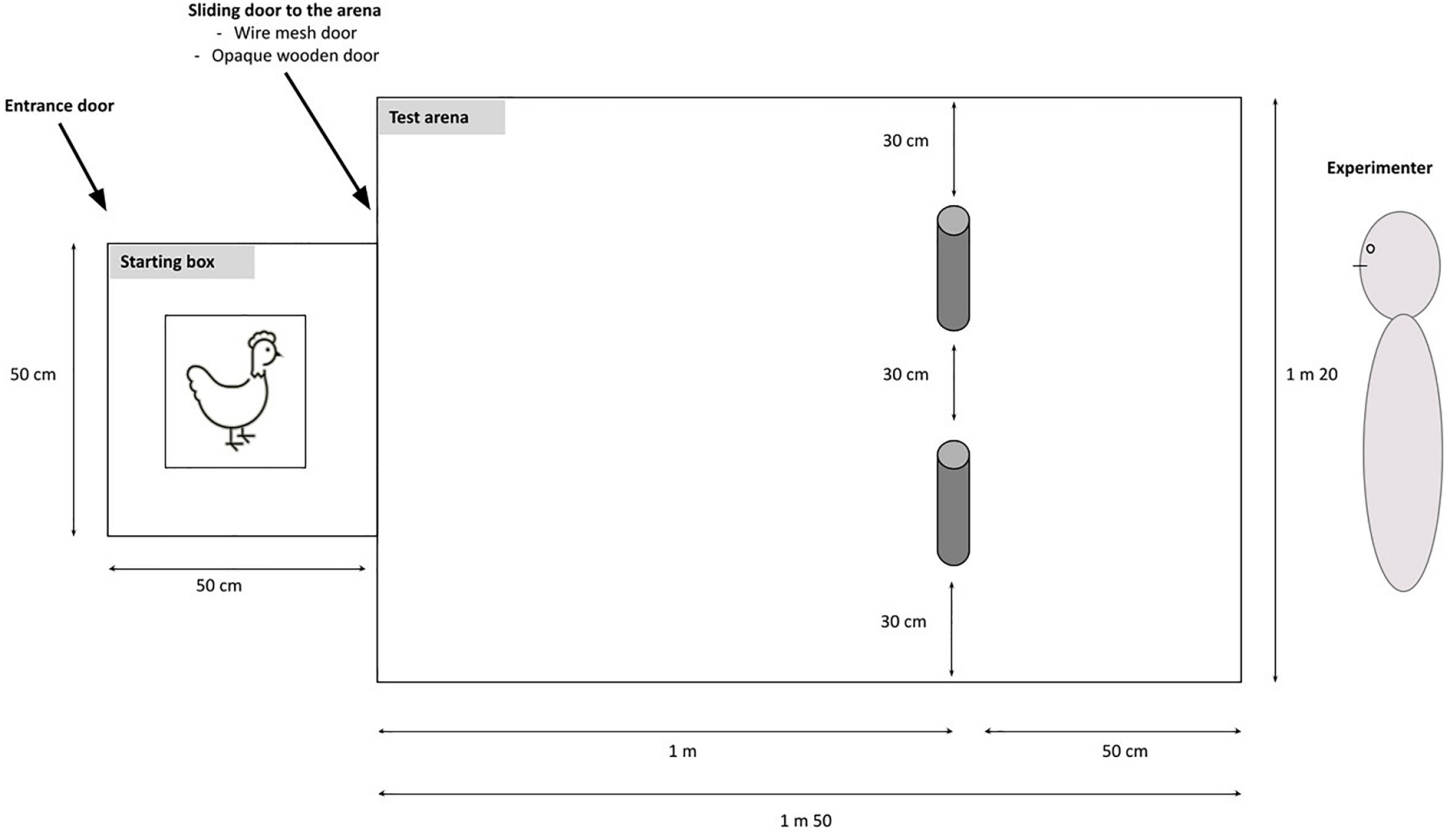
Illustration of the starting box and the test arena.

The test arena was 1.5 m long and 1.2 m large, surrounded by a 40 cm high opaque wooden fence, and covered with a light green PVC floor (Fig. 1). A starting box (69 cm length × 55 cm width × 80 cm high) made of condensed wood was adjacent to the test arena. Two blue hollow tubes (opaque and straight; 15 cm long and 5 cm in diameter) were placed one meter from the starting box and 30 cm from each other. The width of the tubes and their position on the floor were adjusted, to ensure hens would have to lower their head markedly to look inside, and to ensure hens would be able to see the mealworms from the starting box. The tubes’ configuration was determined according to the ongoing trial (see details below).

At the start of each session, a hen was placed in the starting box, from which the experimenter could open a two-step door leading to the test arena. The experimenter stood outside the arena, at one end, and could open the doors remotely. A first opaque wooden door visually separated the starting box from the test arena, allowing the experimenter to set up the configuration of the tubes and the location of the food reward before each trial, out of the sight of the hen. A second wire-meshed door allowed the hen to look inside the test arena. Before each trial, the tubes were systematically manipulated from the experimenter’s left to their right to avoid any perceptual bias through localized auditory cues. To start a trial, the experimenter opened the opaque door and the hen could see the inside of the test arena for 5 s through the wire-meshed door. After these 5 additional seconds, the wire-meshed door was opened and the hen had access to the test arena. The reward was three mealworms, from mealworms killed the same morning in a water bath.

### Habituation and training

#### Habituation

Hens were gradually and individually habituated to the test arena through six habituation trials (two trials per day). In these trials, two mealworms were placed at each corner and in the middle of the arena to promote exploration. Each trial ended 30 s after the individual ate all mealworms, or after 5 min, whichever came first. Then, further habituation trials were run to encourage birds to find mealworms inside two tubes. For these trials, mealworms were placed at both ends of each tube, and the tubes were brought gradually back from the starting box to their final locations in the arena, 1 m from the starting box. Each hen got 10 sessions of two trials per day (for a total of 3 to 10 min per day) in 10 consecutive days. At the end of the habituation phase, all individuals had explored the arena fully and were used to finding mealworms in tubes only, within 30 s. The hens were considered to be accustomed to the testing environment when they had completed the 20 habituation trials and showed no fear behavior (abnormal immobility, movement or vocalizations).

#### Training

Once the hens were accustomed to the testing environment, each hen got training trials in which two mealworms were available only at one end of one of the two tubes. In this phase, the two tubes were arranged 90° from the axis of the waiting cage (standard trials, ST) so that the hen could see the side of the tubes but could not see the inside, and thus the content, of any tube from the starting box. The mealworms were always placed at the outer end of the tube (near the walls) so that the hens were habituated to search for the mealworms from this end. This training prevented the hens from approaching the tubes by positioning themselves between the two, making their choice of one or the other tube clear for the analyses. Each hen got three sessions of 10 training trials with one session per day in three consecutive days. The location of the mealworms (left or right tube) was not the same more than twice in a row, and both locations were presented in equal numbers.

### Testing procedure

#### General procedure

The experiment was divided into three stages. First, 12 hens were tested in free-choice conditions with two hollow tubes for 6 sessions. Second, to investigate further the foraging strategy of the hens, the group was split in two equal cohorts which processed two different tests: either with a different object (square box) in free-choice conditions, or with a more constraining environment in forced-choice conditions (same hollow tubes than first stage). This stage lasted 2 sessions. Finally, for groups that would get a better performance in stage 2 than in the 2 last sessions of stage 1, a third stage was implemented, consisting in further sessions, with the aim to analyze the evolution of the search strategy towards an exclusion performance (a choice by exclusion). The configuration of the sessions in the different stages is detailed below.

**Figure 2:**
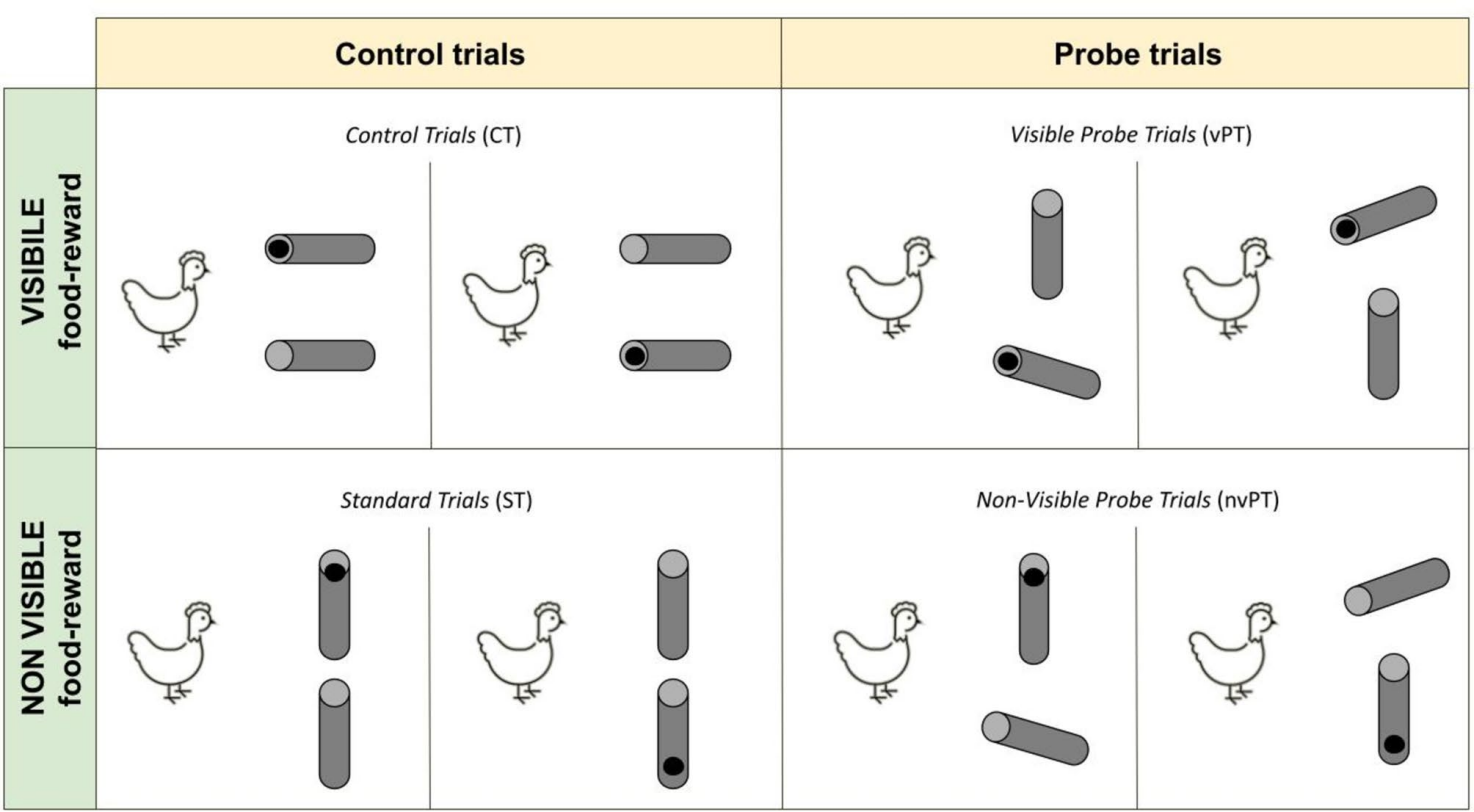
Illustration of the four trial configurations (visible and non-visible food reward). The location of the food reward is represented with a black dot. In control trials (CT), the content of both tubes is visible from the starting box (20°). In standard trails (ST), the content of both tubes is not visible from the starting box (90°). In probe trials, the content of one tube is visible from the starting box but not the other: in visible probe trials (vPT), only the content of the baited tube is visible; in non-visible probe trials (nvPT), only the content of the empty tube is visible. The four trial configurations were used during either of the two testing conditions (free- or forced-choice).

The test sessions were composed of four trial configurations that were distributed differently according to the testing phase/group. For each trial, hens had to find the food reward in one out of two objects. Depending on the testing phase, four trial configurations could be presented (see Fig. 2):

- Visible food reward configurations:
  - Control trials (CT): in these trials, that are positive control trials, hens had the complete information about the location of the food reward: the content of both tubes was visible from the starting box (20°). Hens were expected to perform above chance level and go directly towards the baited tube.
  - Visible probe trials (vPT): in these trials, the content of the baited tube was visible from the starting box (20°), while the content of the empty tube was not (90°). Hens were expected to perform above chance level and go directly towards the easily visible baited tube.
- Non-visible food reward configurations:
  - Non-visible probe trials (nvPT): the inside, and thus the content, of the empty tube was visible from the starting box (20°), while the content of the baited tube was not (90°). In these trials, hens could possibly use the information of the absence of the food reward in one tube to exclude this possibility and choose the other tube (exclusion performance).
  - Standard trials (ST): in these trials, that are negative control trials, hens had no information about the location of the food reward: the content of both tubes was not visible from the starting box (90°). Hens were expected to perform at chance level as they had no indication about the location of the reward. These trials were used as perceptual bias controls.

Mealworms were always placed at the same end of a tube, i.e., either the end close to the starting box if they were in the 20° tube (visible content), or the end close to the arena wall if they were in the 90° tube (non-visible content), according to the trial configuration. Trial configurations and the food reward location were pseudo-randomly determined: a same trial configuration could not occur more than twice consecutively, and the food reward could not be located on the same side (left or right tube) more than twice consecutively.

The targeted behavior recorded was the first tube chosen, that is, the first tube the hen looked inside, touched or pecked at, or the first tube they approached in a 5-cm distance with or without having touched it or looked inside. A look inside a tube was considered when the hen looked inside a tube with mono- or binocular vision by being at 5 cm or less from this tube. As the hens were trained to find the mealworms at the external end of the tubes (near the walls) in non-visible trials, the behavior of positioning themselves between the two tubes to search for the food reward did not occur. A trial was considered correct only when the baited tube was the first tube chosen, as defined earlier. The consequences for an incorrect trial were different among the testing phases and are detailed in the following sections. If the hen did not choose one tube in 3 min, the session was postponed.

#### Sessions structuring

##### Stage 1: spontaneous performance under free-choice condition

Six test sessions were run and included 12 individuals. Each of these sessions contained 10 trials with 4 ST, 2 CT, 2 vPT and 2 nvPT (*M-sessions* with mixed trial configurations).

**Figure 3:**
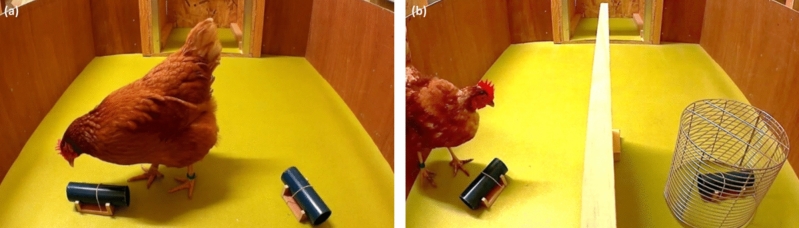
Illustration of the test arena in free-choice and in forced-choice conditions. (**a**) Photograph illustrating the free-choice conditions during a non-visible probe trial (nvPT) with the food reward hidden in the left tube (experimenter’s view). Here, the behavior of the hen is considered as having chosen the left tube (experimenter’s view), as she looked inside it first. The starting box can be seen in the background. (**b**) Photograph illustrating the forced-choice conditions during a non-visible probe trial (nvPT) with the food reward hidden in the right tube (experimenter’s view). A wall divided the arena into two equal parts of 60 × 50 cm. This wall was only used under forced-choice conditions to increase the negative weight of not choosing the baited tube in nvPT trials. After an incorrect trial, i.e., choosing the unbaited tube first, a grid was placed so that the hen could no longer access the other tube, in which the food reward was.

Then, to further modulate the rate of information, we increased the number of nvPT trials per session. Two other sessions (*P-sessions* with only vPT and nvPT) were run for each hen, each comprising 6 nvPT and 6 vPT at each session. A photograph of the test arena during the free-choice condition can be seen in Fig. 3a.

##### Stage 2: testing two different environmental changes

After 8 sessions summing at least 24 trials for each trial type (for statistical convenience), we split the group in two cohorts of six hens each, to test the adaptation of the foraging behavior of the hens under environmental changes. The hens were randomly assigned to the following groups. In group 1, containers with different features were used and the hens were tested under free-choice conditions as before; in group 2, the same containers as before were used (the tubes) but the hens were tested under forced-choice conditions.

**Figure 4:**
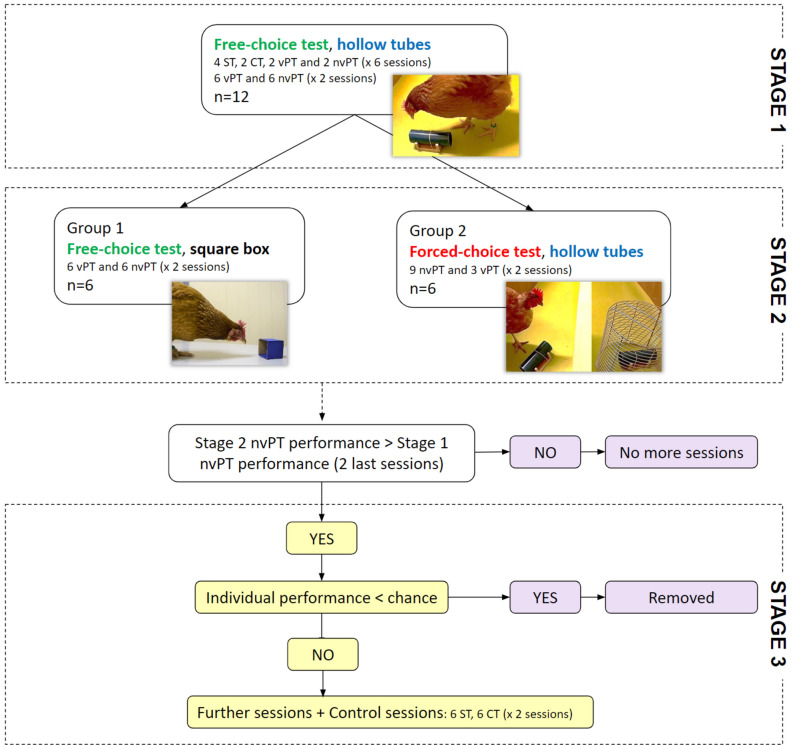
Summary of the steps of the experiment, detailing the number of individuals included at the start of each step, the number of sessions and the distribution of the trials’ configurations at each step. *ST* standard trials, *CT* control trials, *vPT* visible probe trials, *nvPT* non-visible probe trials, *n* number of individuals.

*Group 1: free-choice conditions with a different container (square box)* Trials conducted with the first group of 6 hens aimed to determine whether the foraging behavior of the hens under free-choice conditions was influenced by a change in the container used in the test. The novel containers were two blue 5 cm square boxes with only one opening, compared to the two openings of the initial containers which were tubes (Fig. 4). Four habituation trials were run with one square box to habituate hens to find mealworms in it (2 visible trials and 2 non-visible trials). Then, two test sessions were run, each including 6 vPT and 6 nvPT. The sessions were scheduled as the *P-sessions* (with only vPT and nvPT), so that the results from these two conditions could be compared.

*Group 2: forced-choice conditions with the initial container (hollow tubes)* Trials conducted with the second group of 6 hens aimed to determine whether the foraging behavior of the hens was influenced by constraints on searching conditions (free-choice versus forced-choice conditions). The containers were the initial hollow tubes. Under forced-conditions, an opaque wall (40.5 cm long × 60 cm high) was placed between the two tubes and separated the second half of the arena in two equal sides. To increase the negative weight of not choosing the baited tube in nvPT trials, the sessions mirrored the *P-sessions*, but with more nvPT trials in each session. Two sessions were run, each including 9 nvPT and 3 vPT. Furthermore, a grid was placed over the remaining tube following the initial choice and the hens could therefore no longer access the other tube (Fig. 3b).

##### Stage 3: further sessions

In the case of a better group performance in nvPT trials in stage 2 than in the 2 last sessions of stage 1, for group 1 and/or group 2, a third stage was implemented. This third stage consisted in further sessions, with the aim to analyze the evolution of the search strategy towards an exclusion performance. These sessions were structured as in stage 2 depending on the group (group 1 or group 2). Individuals with a nvPT performance worse than chance in stage 2 were removed from the experiment. Finally, two control sessions were run, each including 6 standard trials (ST) and 6 control trials (CT). Figure 4 summarizes the different steps from the beginning of the experiment to this last step.

### Statistical analysis

A non-parametric approach was used due to the small sample size (n = 12) to ensure a correct statistical power for the analyses. All statistical analyses were performed using R version 4.2.0^32^ with the packages *tidyverse*^33^, *rstatix*^34^, *ggrepel*^35^, *AICcmodavg*^36^, *nlme*^37^ and *lme4*^38^. We considered p-values below 0.05 to be statistically significant for all statistical analyses. Chance level was considered at 50% of success.

Performance was analyzed by comparing the number of successful trials to the total number of performed trials, for each type of trial (CT, ST, vPT and nvPT) and for each testing condition. A trial was considered successful when the baited tube was the first tube chosen, i.e., the first tube the hen looked inside, touched or pecked at, or the first tube they approached in a 5-cm distance with or without having touched it or looked inside. The performance under each testing condition was clustered by 2 sessions to get a relevant number of trials for statistical analysis at the individual level when needed (a minimum of 12 trials).

Two-tailed exact binomial tests were used to test the statistical significance of individual performance per testing condition, and two-tailed Wilcoxon tests were used to assess the statistical significance of the performance at the group level. A Tukey HSD allowed the comparison of the performance between the different conditions tested. Cohen’s d effect size was calculated for each pairwise comparison. For these analyses, the data has been grouped into overall mean performance per individual (all sessions).

We ran generalized linear models to analyze an eventual side bias for the side of the first container chosen (left or right), and its relation to the conditions (free- or forced-choice). The response variable was the side bias, i.e., the side first chosen at each trial. Explanatory variables were the individual, the trial type (CT, ST, vPT or nvPT) and the visibility of the food reward. A comparison of corrected Akaike Information Criterion was used for model selection, if necessary. Kruskal–Wallis tests were run to detail the analysis, followed by Dunn post-hoc tests. Homogeneity of variances was assessed with Levene tests before running multiple comparisons analyses and before model fitting. For model analyses, in order to make more precise analyses of the effect of the fixed parameters on the hens’ behavior (potential side bias, potentially depending on the trial configuration and/or stage), the data has been grouped into mean performance per individual per session as the response variable.

## Results

The results concerning the statistical significance for the different trial configurations at each stage are reported in Supplementary Table SI 1. The individual performances are detailed in Supplementary Table SI 2. Pairwise comparisons for the performance towards a choice by exclusion in nvPT trials, between each stage, and effect sizes, are reported in Supplementary Table SI 3.

### Stage 1: spontaneous performance under free-choice conditions

#### M-sessions

**Figure 5:**
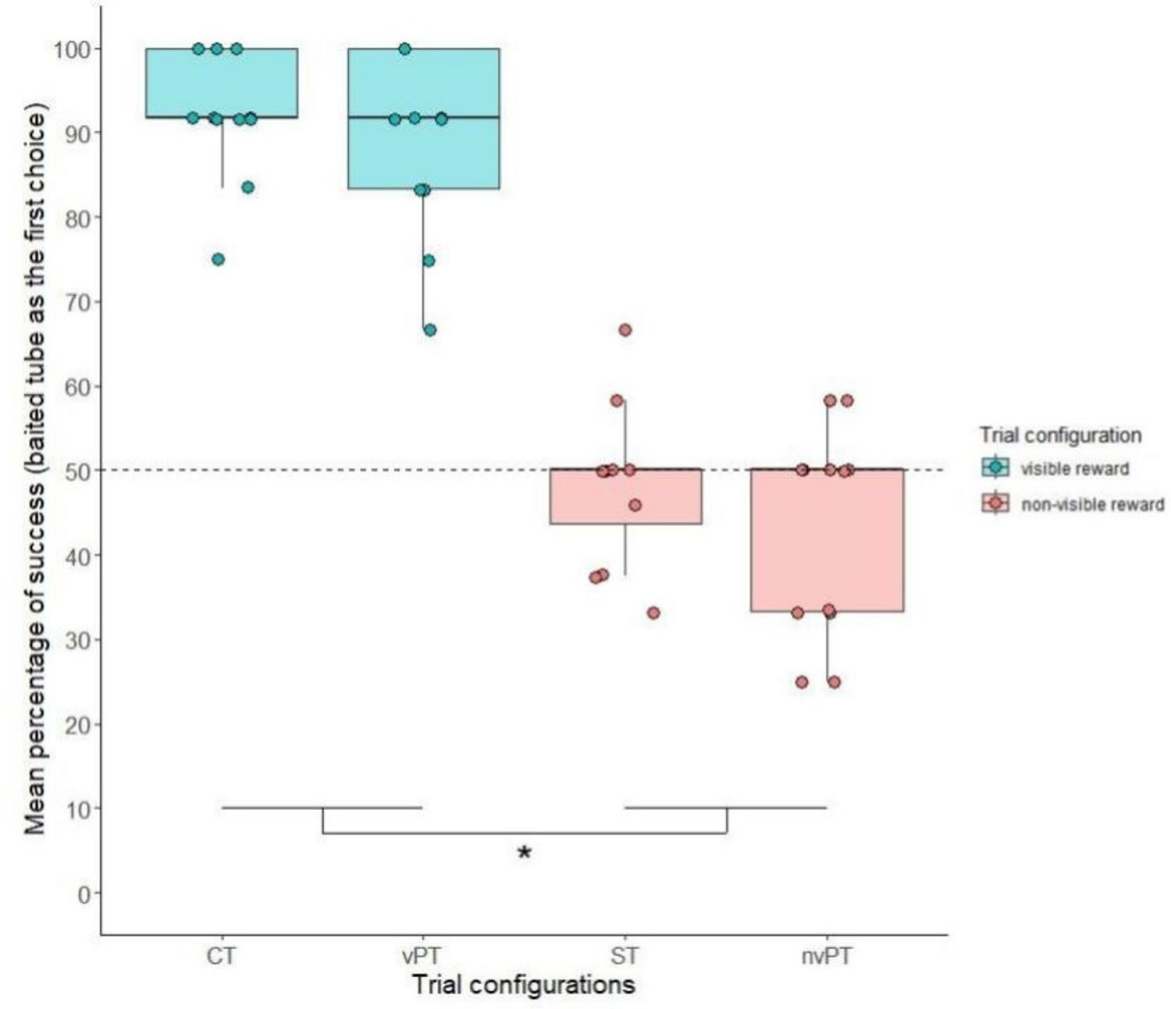
Mean percentage of success (i.e., baited tube chosen first) in the different trial configurations under free-choice conditions. The performance is significantly different between the configurations in which the reward is visible (CT and vPT) and the configurations in which the reward is not visible (ST and nvPT). Each point corresponds to the mean performance per individual per trial configuration. The dashed line corresponds to the 50% level of performance.

In *M-sessions* (mixed trial configurations), the different trial configurations were equally intermixed. The different configurations are reported in Fig. 2. At the group level, the performance for control trials (CT) as well as for visible probe trials (vPT) were significantly greater than chance (mean performances for CT: 92.36 ± 7.50%, V = 78, *p* = 0.0023; and for vPT: 89.58 ± 10.73%, V = 78, *p* = 0.0021) suggesting that the hens differentiated between visible and non-visible conditions. The performance for standard trials (ST) was not significantly different from chance (mean = 48.26 ± 9.14%, V = 7.5, *p* = 0.60) which indicates that the hens had no perceptual indication about the reward’s location. The mean performance for nvPT trials was 43.05 ± 12.22%, at chance level (V = 3, p = 0.073) and all individuals performed at chance level (binomial tests, p > 0.05). The results are illustrated in Fig. 5.

#### P-sessions

In *P-sessions* (with only vPT and nvPT), non-visible probe trials (nvPT) were given at a higher ratio in each session and intermixed with visible probe trials (vPT). At the group level, the mean performance for nvPT was 31.95 ± 12.22%, significantly worse than chance (V = 0, *p* = 0.022). At the individual level, almost all of the individuals performed at chance level (*p* > 0.05) with four individuals performing significantly worse than chance (*p* < 0.05), meaning that the hens tended to choose the tube they could see inside even if it was unbaited. The mean performance for vPT was 94.45 ± 11.42%, greater than chance level (V = 78, *p* = 0.0015), which suggests again that the hens differentiated between visible and non-visible test conditions.

### Stage 2: testing two different environmental changes

#### Group 1: free-choice conditions with a different container (square box)

For group 1, the mean performance for nvPT was 29.17 ± 18.07%, at chance level (V = 0, *p* = 0.057), with a tendency towards being worse than chance. At the individual level, no individual performed better than 50% with two hens performing significantly worse than chance (*France* and *Pearl*). The mean performance for vPT was 95.83 ± 6.97%, above chance level (V = 21, *p* = 0.031).

#### Group 2: forced-choice test with the tubes

For group 2, the mean performance for nvPT was 42.59 ± 25.50%, at chance level (V = 3, *p* = 0.58). At the individual level, four hens performed at chance level and two hens performed worse than chance (*Starr* and *Soleil*). The mean performance for vPT trials was 97.22 ± 6.81%, above chance level (V = 21, *p* = 0.026).

### Stage 3: further sessions

Further sessions were programmed with the aim to study further the modalities of adoption of a choice by exclusion by hens, focusing on nvPT performance. The group nvPT performance in Group 1 (new container: square box) did not improve compared to the 2 last sessions of Stage 1; however, the group nvPT performance in Group 2 (new constraints: forced-choice conditions) did improve. Thus, we tested Group 2 individuals with further sessions. In this group, two individuals (*Starr* and *Soleil*) were removed as they showed a performance significantly worse than chance in stage 2 (binomial tests, *p* < 0.05). Due to the small sample size (n = 4), the results at the group level are presented in a descriptive manner.

**Figure 6:**
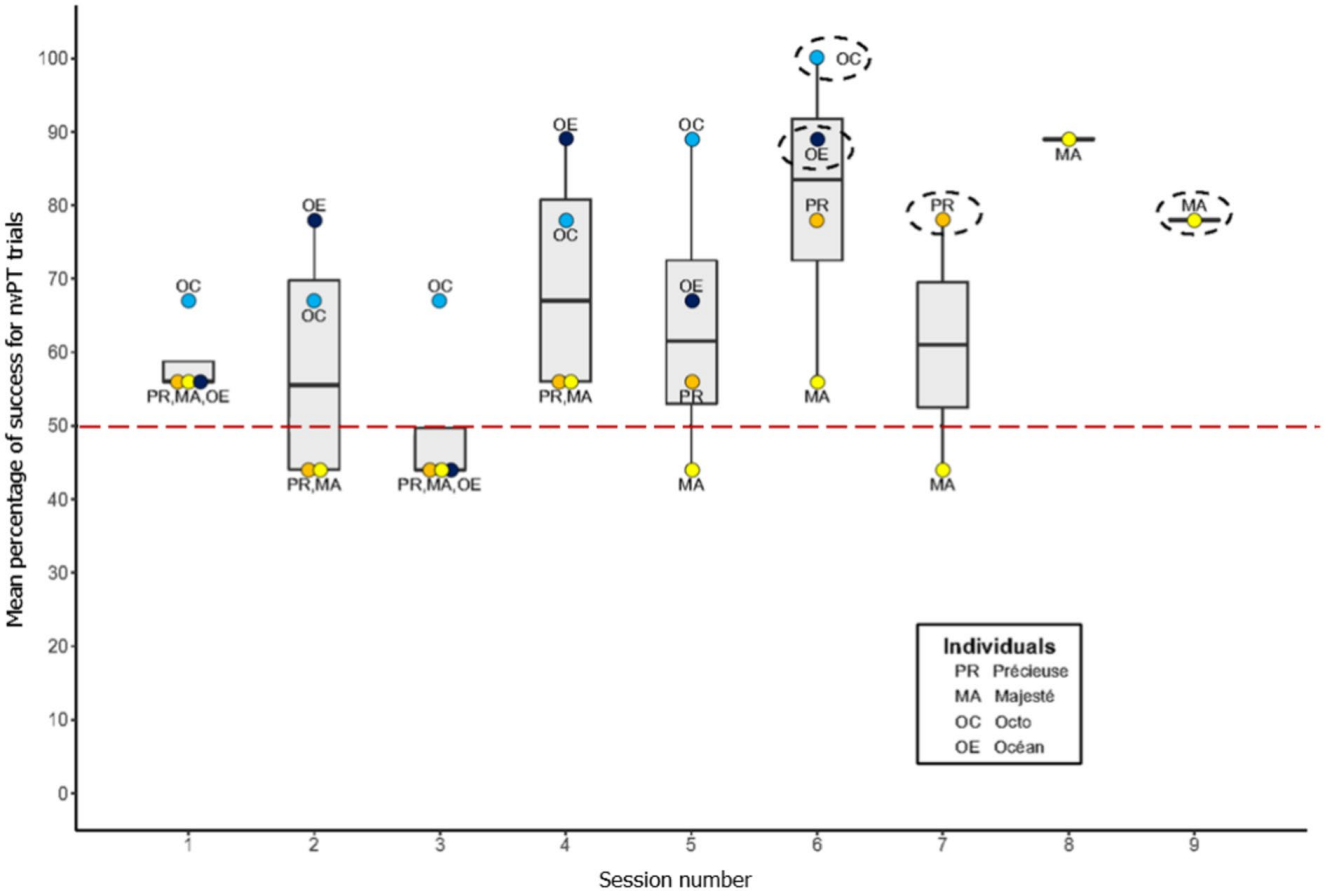
Evolution of individuals’ performance in nvPT trials under forced-choice conditions, for each session. To reach a performance significantly better than chance, individuals had to validate an exclusion performance with at least 14 trials out of 18 (2 consecutive sessions, binomial test). The circled points correspond to the session of validation, for each individual. The dashed line corresponds to the 50% level. For each boxplot, dots are the mean individual performances, vertical lines are standard deviation, and the horizontal line shows the median value.

Throughout further sessions under forced-choice conditions, the performance of the four individuals increased in nvPT trials (mean performance at the group level: first two sessions = 58.33 ± 9.62%; last two sessions for each individual = 83.5 ± 7.78%). Their performance throughout the sessions can be seen in Fig. 6. The four individuals performed greater than chance (at least 14/18 successful nvPT summing the last two consecutive sessions; binomial tests per individual with *p* < 0.05) respectively in 6 sessions (nvPT performance for the 2 last sessions: *Octo*: 94.5% and *Océan*: 78%), 7 sessions (*Précieuse*: 78%) and 9 sessions (*Majesté*: 83.5%). The performance on the last two sessions was better than the group performance in any other tested condition (Supplementary Table SI 3). The mean performance for vPT trials in the last two sessions was 91.62 ± 9.62%, above chance level (n = 4; binomial tests per individual with *p* < 0.05).

Finally, control sessions were run for these four individuals, to control for perceptual cues at this stage. These sessions included control trials (CT, complete information about the location of the reward) and standard trials (ST, no information). The group performance in CT was greater than the chance level (mean = 100 ± 0%, n = 4; binomial tests per individual with *p* < 0.05) meaning hens differentiated between visible and non-visible conditions. The group performance in ST was at chance level (mean = 52.08 ± 4.17%, n = 4; binomial tests per individual with *p* > 0.05) suggesting hens had no perceptual indication about the food reward’s location when they did not see it.

### Side bias under free-choice conditions

**Figure 7:**
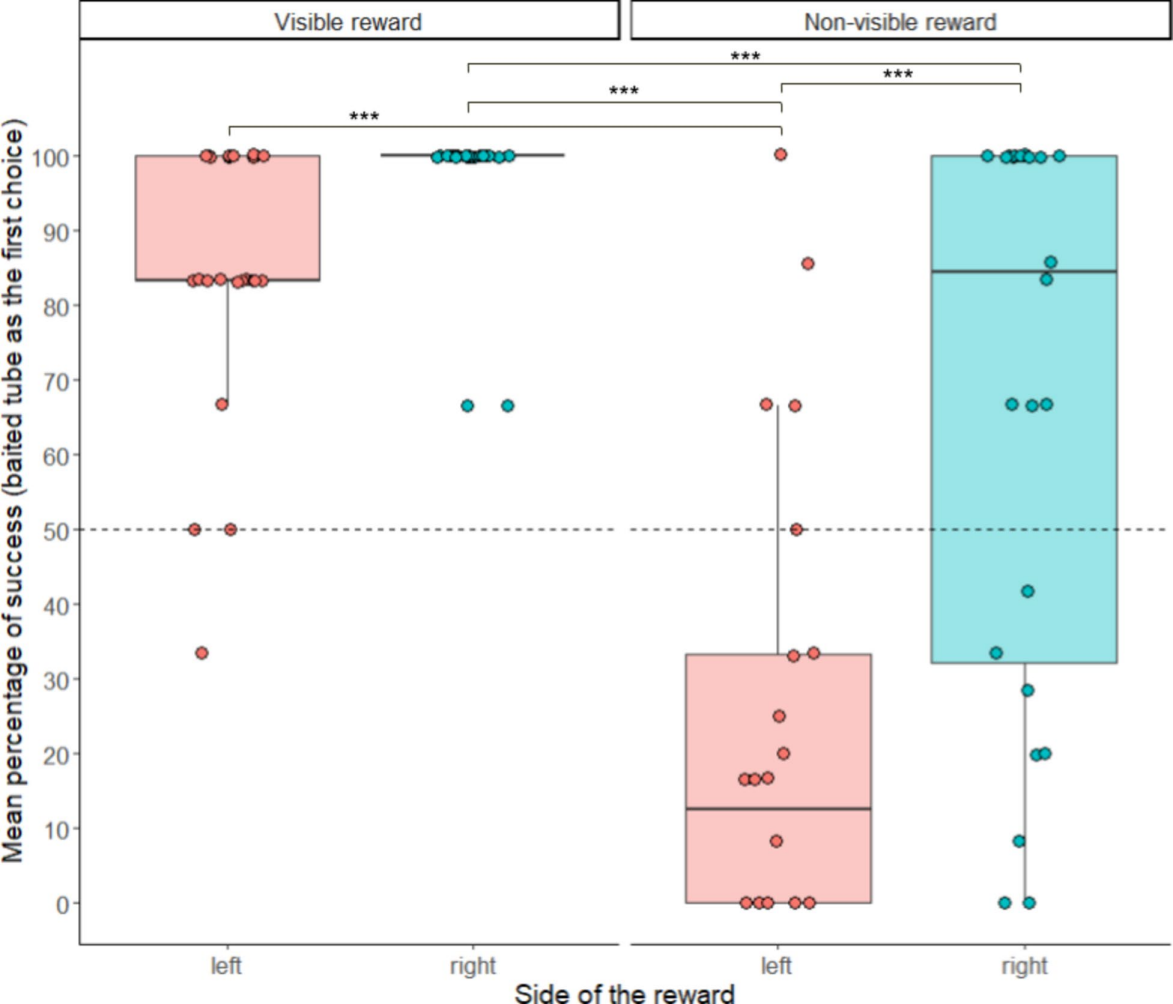
The container first chosen was significantly biased towards the right side in the configurations in which the reward could not be directly visible from the starting point (ST and nvPT). The comparisons between each result (visibility of the reward × side reward) was significantly different (p < 0.001) except for the conditions visible reward, left—non-visible reward, right (p = 0.101) and visible reward, left—visible reward, right (p = 0.336). Each point corresponds to the performance per session per individual. The dashed line corresponds to the 50% level of performance. ***p < 0.001.

At the group level, we found a significantly biased choice for one side, depending on the trial configuration (KW Chi2 = 17.68, *p* < 0.001, Supplementary Table SI 4). The strength of the side bias depended on the individual (KW Chi2 = 162, *p* < 0.001, SI 4). More precisely, there was significant differences for the side bias between CT and nvPT (*p* = 0.0043), between CT and ST (*p* = 0.0035), and between vPT and ST (*p* = 0.0041), with a right bias in nvPT and ST, but no side bias in CT and vPT (Supplementary Fig. SI 7). Indeed, the visibility of the reward (i.e., whether the content of the baited container was visible or not) was a better predictor of the side bias than the trial configuration (dAICc = 3.68, Supplementary Tables SI 5, SI 6). There was a significant difference between visible (CT and vPT) and non-visible (ST and nvPT) trials, showing a stronger right bias in non-visible trials (Kruskal–Wallis Chi2 = 17.42, *p* < 0.001; see Fig. 7). This result was also found in *P-sessions* (stage 1, z = 3.150, *p* = 0.0016) but not in Group 1 sessions (stage 2, *p* = 0.080).

## Discussion

The main objective of this study was to investigate how domestic hens adapt their foraging strategy in a two-choice task, as a function of the information available to reach the location of the food reward (complete, partial, or no information), and on environmental conditions (with the tubes or with the boxes; free-choice or forced-choice conditions). Overall, our results suggest that the hens applied an optimal strategy according to the information available on the location of the food reward under free-choice conditions; and that some individuals were able to adapt by choosing by exclusion, over several sessions when the risk of not obtaining the food reward was higher, i.e., under forced-choice conditions.

Under free-choice conditions, the hens applied an optimal strategy according to the information available on each trial. In trials where the food was directly visible (CT and vPT), the hens remained attentive and retrieved the food directly. In trials where the food was not visible (ST and nvPT), the hens systematically applied a side-biased strategy, moving first to the tube to their right, which led to a random performance. As they were tested under free-choice conditions, they were not penalized regardless of the first choice they made. In this type of random choice task, where both options are equally rewarding^39^, choosing one of the two options and sticking to it is a common speed-accuracy trade-off^40,41^.

The side-biased strategy when no information is available could be linked to the behavioral ecology of the species. Indeed, in some species such as poultry, exploratory behaviors such as the “search until you find” strategy^42^ are associated with a greater chance of finding food in natural conditions^30^. The hens’ bias towards the right side during free-choice conditions could be explained by the fact that, by choosing the container on their right side, hens had visual access to the food with the left eye. A study from Regolin et al.^43^ on chicks suggests that the right hemisphere, where the neural information from the left eye is projected contralaterally, may be responsible for spatially targeted foraging. Thus, the rightward bias we observed could be due to the foraging principle of the task^44,45^.

In nvPT trials under free-choice conditions, the hens could have used the information of the absence of the food reward in a tube to adopt an exclusion strategy, but they did not do it. The group performance reached 43% of success in the first twelve trials (*M-sessions*, with mixed trial configurations) and dropped to 32% in further trials (*P-sessions*, sessions with only vMT and nvPT). Lack of motivation could not have led to this result as hens always responded correctly during visible trials, i.e., they moved towards the reward when they could see it (vPT and CT). It is also unlikely that tube features could have influenced the birds’ performance as performance was not improved in nvPT trials with a container with different features (square box, group 1). This result rather suggests that, in further nvPT trials, hens tended to choose the container whose content was visible, even if it was empty. The propensity persisted whatever the type of container, which did not affect their search strategy (group 1, group performance for nvPT trials of 29%). This tendency to prefer containers whose inside is visible has already been highlighted in chicks^46^. The present study seems to confirm this behavior in adult hens.

For group 2 under forced-choice conditions, we were able to test for the adaptability of the hens’ foraging strategy in a more constraining environment, where they were no longer free in their search strategy. In nvPT trials under forced-choice conditions, in the two first sessions (18 nvPT trials, group 2), the group performance reached 43% of successful trials and three hens were successful at their very first nvPT trial. This performance is equal to that of the group under free-choice conditions (43% of success during the first twelve trials), which means that the hens were able to adapt quickly to the new rules. This is a good performance compared to other analogous studies in corvids^19,22^ in which the group performance reached a maximum of 19.17% of success, in forced-choice trials for which exclusion could have been applicable (but with different containers). However, it should be noted that a great inter-individual variation was found in the hens’ performance (minimum of 11% and maximum of 67% of success among individuals), with two individuals having obtained results below chance. This result highlights the need to take individual traits into account, and calls for a better understanding of how domestic birds adapt their behavior according to different environmental constraints, including the farming environments.

Under forced-choice conditions, four out of six hens learned the exclusion rule over several sessions: they reached a performance significantly above chance level in 6 to 9 sessions (i.e., within 36 to 56 trials). This result is supported by the fact that the hens adapted their search strategy according to the trial configuration. Previous studies have shown that some birds are able to choose by exclusion (mainly corvids and parrots^22–26^; ground-hornbills^27^, skuas^28^, pigeons^29^) but this behavior has not yet been demonstrated in chickens. This exclusion performance, as discussed in the literature^26,47^, can rely on cognitive processes by inference or more simply by learning to avoid the unbaited container. Further investigations are needed to unravel the cognitive processes underlying hens’ exclusion performance.

The exclusion performance of the hens (group level; non-visible probe trials) increased only under forced choice condition, while it decreased in free-choice condition (whether with the hollow tubes or the square box). This result suggests that forced-choice conditions, compared to free-choice conditions, could be more likely to favor the adoption of an exclusion-based foraging strategy in hens. At this point, it is difficult to fully explain this result, however, it is known that forced-choice tasks improve early attentional processes and facilitate perceptual processing of stimuli involved in the early decisional process^48^. Otherwise, it must be pointed out that the simple risk of not obtaining the reward under forced-choice conditions was sufficient in itself for four out of six hens to learn a more effective strategy, with no food deprivation nor further negative reinforcement.

This study is a first proof of concept of the flexibility of the different search strategies that can be adopted by hens in foraging. It should be approached in the light of its main limitations, that are the small sample size and the limited generalization to population, as the hens were from the same laying strain and were kept in the same living environment. The replication of such study with other strains and in different living environments would be of value to support the present results.

In conclusion, the domestic hens applied an optimal foraging strategy depending both on the available information (visible or non-visible food reward) and the external environmental constraints (free- or forced-choice conditions). They were able to adapt their search behavior to the information available at each trial, and, for some individuals, to switch to an exclusion performance under forced-choice conditions. Through a cognitive approach, this study contributes to a better understanding of the foraging strategies in domestic hens and could pave the way to practical, foraging-based enrichment solutions in order to improve their welfare in farming systems.

### Supplementary Information

Supplementary Information.

Supplementary Figure 7.

## Data Availability

The dataset generated and analyzed during the current study is hosted in the INRA data repository at https://entrepot.recherche.data.gouv.fr/privateurl.xhtml?token=fd703b27-d819-43d9-b610-f431c9f4f161. The dataset is available on reasonable request from the corresponding author at rachel.degrande@gmail.com.
