# Supplementary material for: A cognitive approach to better understand foraging strategies of the adult domestic hen
Source: Sci Rep. 2024 Aug 20;14:19265. doi: 10.1038/s41598-024-70093-3 (PMC11336211; doi:10.1038/s41598-024-70093-3)

Supplementary Materials ESM-1

A cognitive approach to better understand foraging strategies of the adult domestic hen

Degrande R. [a] *, Cornilleau F. [a], Jardat P. [a], Ferreira V.H.B. [a], Lansade L. [a], Calandreau L. [a]

[a]CNRS, IFCE, INRAE, Université de Tours, PRC (Physiologie de la Reproduction et des Comportements), F-37380, Nouzilly, Indre-et-Loire, France

* Corresponding author: rachel.degrande@gmail.com

SI 1: Mean performance +/- SD (%) for each trial type at each stage. For each line, the pvalue from the comparison with the chance level is detailed (Wilcoxon test). • = statistical tendencies (*P* < 0.1) considered when tested individuals are four.

Stage	Trial type	Mean (%)	Sd (%)	P (vs 50%)
Free-choice test, *M-sessions*	vPT	89.58	10.73	0.0023
	nvPT	43.05	12.22	0.073
	CT	92.36	7.5	0.0021
	ST	48.26	9.14	0.60
Free-choice test, *P-sessions*	vPT	94.45	11.42	0.0015
	nvPT	31.95	12.22	0.022
Group 1: Different object	vPT	95.83	6.97	0.031
	nvPT	29.17	18.07	0.057
Group 2: Forced-choice test (2 first sessions)	vPT	97.22	6.81	0.026
	nvPT	42.59	25.50	0.58
Group 2: Forced-choice test, final sessions	vPT	91.67	9.62	0.095 •
	nvPT	83.33	7.85	0.098 •
Forced-choice test controls	CT	100	0	0.072 •
	ST	52.08	4.17	1 •

SI 2: Mean percent of success detailed per individual at each stage, for each session type and each trial type. *: vPT and nvPT results are not detailed here as they consist of a number of sessions until reaching a performance better than chance level in the two last consecutive sessions.

				Trial type			
Stage	Session type	id	Individual	CT	vPT	ST	nvPT
Stage 1	M-sessions	EL	Elizabeth	91,67	83,33	66,67	50,00
		SA	Savana	83,33	83,33	37,50	58,33
		RO	Rouge	91,67	91,67	50,00	58,33
		FR	France	91,67	91,67	45,83	33,33
		PE	Pearl	100,00	100,00	50,00	33,33
		DA	Daenerys	91,67	91,67	58,33	33,33
		OE	Ocean	75,00	66,67	50,00	50,00
		PR	Précieuse	100,00	100,00	50,00	50,00
		OC	Octo	91,67	75,00	50,00	50,00
		SO	Soleil	100,00	100,00	33,33	25,00
		MA	Majesté	91,67	91,67	50,00	50,00
		ST	Starr	100,00	100,00	37,50	25,00
	P-sessions	EL	Elizabeth		66,67		50,00
		SA	Savana		100,00		16,67
		RO	Rouge		100,00		50,00
		FR	France		100,00		0,00
		PE	Pearl		100,00		41,67
		DA	Daenerys		100,00		41,67
		OE	Ocean		91,67		50,00
		PR	Précieuse		100,00		25,00
		OC	Octo		75,00		50,00
		SO	Soleil		100,00		8,33
		MA	Majesté		100,00		50,00
		ST	Starr		100,00		0,00
Stage 2	Group 1	EL	Elizabeth		100,00		50,00
		SA	Savana		100,00		25,00
		RO	Rouge		100,00		41,67
		FR	France		91,67		8,33
		PE	Pearl		83,33		8,33
		DA	Daenerys		100,00		41,67
	Group 2	OE	Ocean		100,00		66,67
		PR	Précieuse		100,00		50,00
		OC	Octo		100,00		66,67
		SO	Soleil		100,00		11,11
		MA	Majesté		83,33		50,00
		ST	Starr		100,00		11,11
Stage 3: control sessions *	Group 2	OE	Ocean	100,00		50,00	
		PR	Précieuse	100,00		50,00	
		OC	Octo	100,00		58,33	
		MA	Majesté	100,00		50,00	

SI 3: Result for the post-hoc comparisons between each tested condition with a Tukey HSD post-hoc test, with the absolute value of Cohen's d effect size for each comparison. Performance in non-visible probe trials (nvPT) was calculated over 2 sessions for each tested condition to get a relevant number of trials for the analysis (i.e. at least 12 trials). Cohen's d is commonly described as weak at and under 0.2, mean around 0.5 and strong at and over 0.8.

**Test conditions compared**		**Difference**	**Lower**	**Upper**	**Adjusted *P*-value**	**Effect size (abs. Cohen's d)**
**Forced-choice, 2 last sessions**	Forced-choice, 2 first sessions (group2)	-40.74	-74.25	-7.23	0.01	1.78
	Free-choice, *M-sessions*	-40.28	-70.25	-10.31	< .0.001	3.33
	Free-choice, *P-sessions*	-51.39	-81.36	-21.41	< .0.001	2.60
	Free-choice with another object (group1)	-54.17	-87.68	-20.66	< .0.001	3.25
**Forced-choice, 2 first sessions**	Free-choice, *M-sessions*	0.46	-25.50	26.42	1.00	0.025
	Free-choice, *P-sessions*	-10.65	-36.61	15.31	0.76	0.455
	Free-choice with another object (group1)	-13.43	-43.40	16.55	0.70	0.561
**Free-choice, M-sessions**	Free-choice, *P-sessions*	-11.11	-32.30	10.08	0.56	0.632
	Free-choice with another object (group1)	-13.89	-39.84	12.07	0.55	0.924
**Free-choice, P-sessions**	Free-choice with another object (group1)	-2.78	-28.74	23.18	1.00	0.133

SI 4: Analysis of the effect of the individual identity and the trial type on the side chosen first, at each trial, during the *M-sessions* in free-choice conditions. Both the individual identity and the trial type have a significant impact on the side chosen first. The generalized mixed model table is not detailed (median of deviance residuals = -0.51). The results of post-hoc Kruskal-Wallis are detailed for the variable Individual and the variable Trial type. The Dunn post-hoc test (Holm correction) is detailed to compare the side chosen first according to the trial type.

**Kruskal-Wallis rank sum test**	**Chi2**	**df**	**p-value**
Side chosen ~ Individual	162	11	<0.001
Side chosen ~ Trial type	17.68	3	<0.001

Dunn test with Holm correction

**Group 1**	**Group 2**	**statistic**	**Adjusted p-value**
CT	nvPT	-2.86	0.020
	vPT	-0.490	1
	ST	-3.44	0.0035
nvPT	vPT	2.37	0.054
	ST	-0.141	1
vPT	ST	-2.87	0.020

SI 5: Model selection for the effect of the trial type (ST, nvPT, vPT and ST), the visibility of the reward (visible or non-visible), of the presence of a tube with a visible content in the trial (CT, vPT or nvPT) or not (ST). The more accurate model has the smallest corrected Akaike Information Criterion value.

**AICc model selection**	**K**	**AICc**
Side chosen ~ trial type + individual	15	761.35
Side chosen ~ reward_visible + individual	13	757.67
Side chosen ~ content_visible + individual	13	770.13

SI 6: The generalized linear model table for the effect of the side of the reward and the visibility of the reward on the performance at each trial, whatever the trial type (individuals as random effects, binomial family).

**Random effects**	**Variance**	**Std. Dev.**
Individuals	0.23	0.48

**Fixed Effects**	**Estimate**	**Std. Error**	**Z value**	**Pr(<|z|)**
Intercept	0.77	0.17	4.46	<0.001
Side of the reward	-2.16	0.15	-14.05	<0.001
Visibility of the reward	3.46	0.20	17.10	<0.001

<u>SI Figure 7</u><u>: Detail of the number of time individuals choose to search the reward on one side or the other (percentage of success) depending on the trial type, the test condition, at the group level.</u> There was a significant effect of the trial type on the side chosen first (*P*<0.05 between CT-nvPT, CT-ST and vPT-ST), but no effect of the test condition. Thus, there was an effect of the side of the reward location on the performance, with a significant side bias for right over left in nvPT and ST but no significant difference in CT and vPT. For each boxplot, dots are the mean individual performances, vertical lines are standard deviation, and the horizontal line shows the median value.

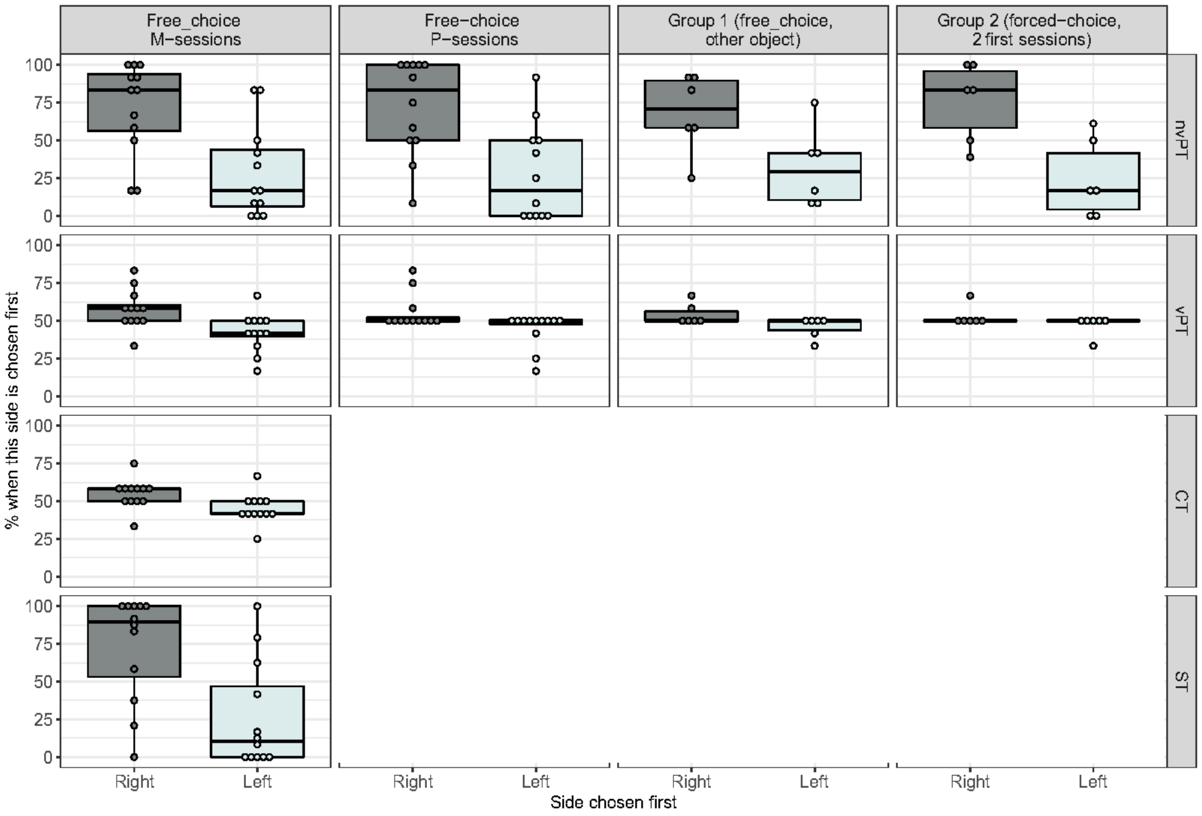

Supplement: Supplementary file 1 — Supplementary Information. [file 41598_2024_70093_MOESM1_ESM.pdf]
