# Supplementary figures and images for: A cognitive approach to better understand foraging strategies of the adult domestic hen
Source: Sci Rep. 2024 Aug 20;14:19265. doi: 10.1038/s41598-024-70093-3 (PMC11336211; doi:10.1038/s41598-024-70093-3)

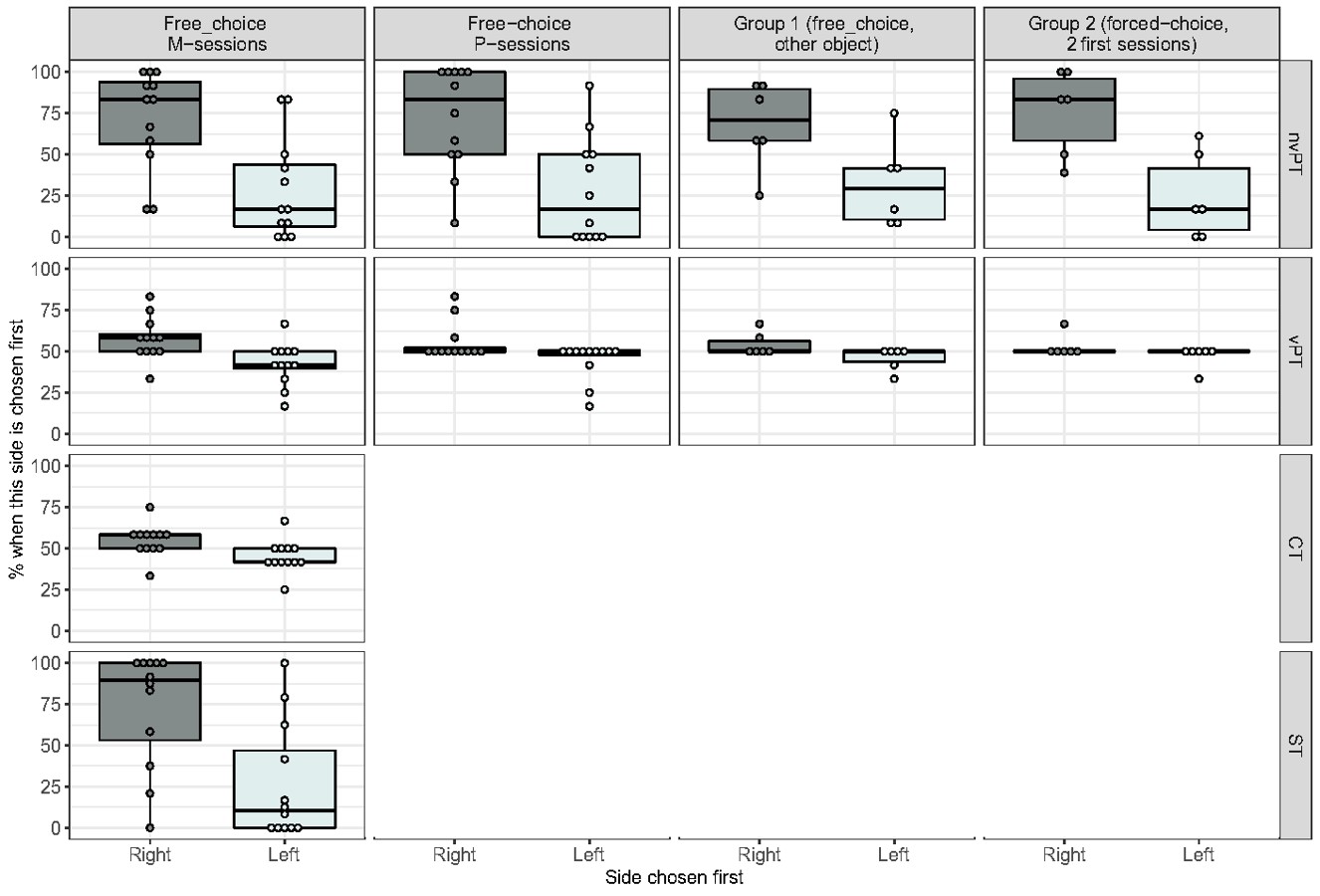

Supplement: Supplementary file 2 — Supplementary Figure 7. [file 41598_2024_70093_MOESM2_ESM.jpg]
